# Limited Scope of Shorter Drug Regimen for MDR TB Caused by High Resistance to Fluoroquinolone

**DOI:** 10.3201/eid2509.190105

**Published:** 2019-09

**Authors:** Pravin K. Singh, Amita Jain

**Affiliations:** King George Medical University, Lucknow, India

**Keywords:** shorter regimen, multidrug-resistant tuberculosis, MDR TB, tuberculosis, TB, tuberculosis and other mycobacteria, bacteria, second-line drugs, antimicrobial resistance, fluoroquinolone, rifampin, line-probe assay, respiratory infections, Uttar Pradesh, India

## Abstract

Resistance to second-line tuberculosis drugs for patients with multidrug-resistant tuberculosis has emerged globally and is a potential risk factor for unfavorable outcomes of shorter duration drug regimens. We assessed the proportion of patients eligible for a shorter drug regimen in Uttar Pradesh, India, which had the highest rate of multidrug-resistant tuberculosis in India.

India has the largest burden of multidrug-resistant (MDR) tuberculosis (TB) worldwide (*1*). The success rate for MDR TB treatment is low (47%), largely caused by death, suboptimal adherence of patients to long treatment courses, and frequent drug-related adverse events (*2*).

In 2016, the World Health Organization recommended a shorter drug regimen (9–12 months) for patients with MDR TB or rifampin-resistant TB who had not received second-line drugs (SLDs) and in whom resistance to fluoroquinolones and injectable SLDs is considered highly unlikely (*3*). A shorter regimen is a promising step toward high treatment success rates. Recently, this regimen was instituted in Uttar Pradesh, which has ≈20% of the total MDR TB and rifampin-resistant TB burden in India (*2*). We assessed the proportion of rifampin-resistant TB patients in Uttar Pradesh who would be eligible for a shorter regimen under a programmatic setting.

Under the Revised National Tuberculosis Control Program for India, all TB patients are tested for drug susceptibility for at least rifampin by the GeneXpert MTB/RIF assay (http://www.cepheid.com), which is available in every district laboratory. Two samples per patient are usually collected, and if rifampin-resistant TB is confirmed, the second sample is transported to the state laboratory for susceptibility testing of SLDs by line-probe assay (LPA) (LPA-SLD Genotype MTBDRsl; Bruker, https://www.hain-lifescience.de). Because it might take ≈1 week to obtain an LPA-SLD result, a shorter treatment regimen is initiated without delay for all patients with rifampin-resistant TB, except for patients who have received SLDs, those with extrapulmonary TB, and those who are pregnant (*4*). If resistance to SLD is detected, patients are given an appropriate, longer treatment regimen.

Over a 2-month period (July–August 2018), sputum samples from 708 patients with rifampin-resistant TB (1 sample/patient) were collected before treatment with any SLDs and submitted to our reference laboratory for LPA-SLD. Smears from each sputum sample were examined for acid-fast bacilli (AFB). AFB-positive samples were subjected directly to LPA, AFB-negative samples were cultured in *Mycobacterium* growth indicator tube liquid medium, and LPA was performed indirectly for culture isolates, if recovered. LPA testing on smear-positive sputum samples with inconclusive results (*Mycobacterium tuberculosis* not detected/indeterminate resistance/invalid) was repeated by using the indirect method. Resistance patterns for fluoroquinolones and injectable SLD (as determined by LPA-SLD) were analyzed.

Of 708 samples, drug susceptibilities were determined for 541 (498 by direct LPA and 43 by indirect LPA). For the remaining 167 samples (AFB negative, 112; AFB scanty, 52), results were inconclusive because of no growth or contamination (Figure). Plausible reasons for lower rate of culture recovery include testing of only AFB-negative/scanty samples, expected low culture yield if patients have a history of tuberculosis treatment or if a sample was of suboptimal quality (*5*), and a harsh decontamination process used for the sample. This last reason is unlikely because the culture contamination rate was not lower than standard range.

**Figure:**
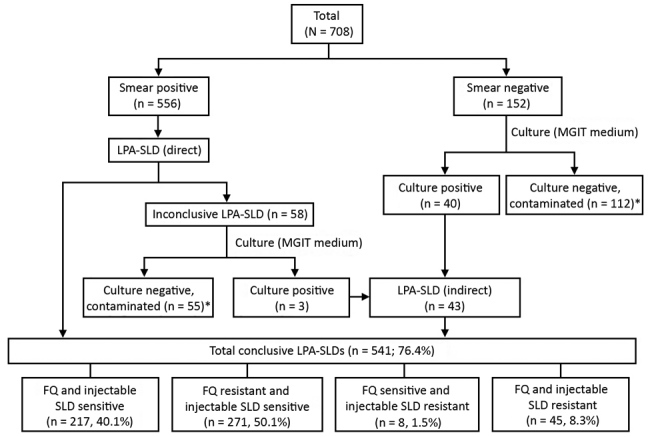
Flow diagram of participants and testing results in study of limitation of shorter treatment regimen for multidrug-resistant tuberculosis by high resistance to fluoroquinolone, Uttar Pradesh, India. *Inconclusive LPA-SLD (n = 167; 23.6% of total samples received). LPA-SLD, line-probe assay for second-line drugs; FQ, fluoroquinolones; MGIT, *Mycobacterium* growth indicator tube.

In our study, a high proportion (21.4%) of patients were smear negative, although they were given a diagnosis of rifampin-resistant TB by GeneXpert. Low analytical sensitivity for smear microscopy (compared with that for GeneXpert) could be a potential reason. This observation highlights the need of submitting 2 quality samples for LPA.

Of 541 conclusive LPA-SLD results, the proportion of strains resistant to only fluoroquinolone was 50.1%, to injectable SLD 1.5%, and to fluoroquinolone and injectable SLD 8.3%. Fluoroquinolone resistance (with or without injectable SLD resistance) was high, indicating that 58.4% of patients were ineligible for a shorter regimen. This estimate raises concern because, in an earlier trial of a shorter regimen, fluoroquinolone resistance was a key risk factor for a bacteriologically unfavorable outcome (*6*).

Our estimate might not be a true representation for fluoroquinolone resistance burden because we included only rifampin-resistant TB patients in our study, not patients with rifampin-susceptible but fluoroquinolone-resistant TB. Nevertheless, our estimate might be extrapolated for a programmatic setting in Uttar Pradesh because the number of patients in the study was ≈7.0% of MDR TB and rifampin-resistant TB patients reported in Uttar Pradesh during 2017 (*1*).

In India, a high incidence of fluoroquinolone-resistant TB has been reported (*7**,**8*). It is believed that widespread and indiscriminate use of fluoroquinolones was a key contributor. However, primary resistance caused by transmission of fluoroquinolone-resistant strains might be a potential reason for such a high rate of fluoroquinolone resistance. This hypothesis is well supported by increasing evidence for countries with high burdens of TB where MDR TB is increasing in patients who have not received TB treatment (*9**,**10*). In our study, we could not stratify our data into new and previously treated patients because of the observational design and limited access to patient information.

Considering high levels of fluoroquinolone resistance and possible intolerance/resistance to certain drugs in a regimen, only ≈40.0% of rifampin-resistant TB patients would be eligible for a shorter drug regimen in our setting. Thus, a shorter regimen should not be an obvious choice for empiric treatment of MDR TB or rifampin-resistant TB patients in this region.
